# Magnetization and magneto-transport staircaselike behavior in layered perovskite Sr_2_CoO_4_ at low temperature

**DOI:** 10.1038/srep27712

**Published:** 2016-06-13

**Authors:** Qiuhang Li, Xueping Yuan, Lei Xing, Mingxiang Xu

**Affiliations:** 1 Department of Physics and Key Laboratory of MEMS of the Ministry of Education, Southeast University, Nanjing 210096, China; 2 Department of Fundamental Courses, Nantong Polytechnic College, Nantong 226002, China

## Abstract

Polycrystalline layered perovskite Sr_2_CoO_4_ sample was synthesized by high temperature and high pressure method. The staircaselike behavior has been observed in the magnetization and resistivity versus field curves of Sr_2_CoO_4_ at low temperature. The main features of the steps can be obtained from the measured results: (i) the positions of the external magnetic field at which steps occur are varying in different measurement runs, (ii) the steps only appear at low temperature and disappear with a slight increase of the temperature, (iii) the steps are dependent on the temperature and field sweep rate. Based on the features of the magnetization and magneto-transport staircaselike behavior in Sr_2_CoO_4_, the unusual phenomenon can be ascribed to an avalanche of flipping domains in terms of the random field theory.

In recent years, two dimensional compounds with K_2_NiF_4_-type structure (a type of tetragonal structures) have generated great interest after the discovery of superconductivity, magnetoresistance (MR), spin/charge stripes in nickelates and manganites^1^,^2^,^3^,^4^,^5^,^6^,^7^,^8^,^9^,^10^,^11^,^12^,^13^. Two-dimensional layer structured perovskite compound Sr_2_CoO_4_ is one of K_2_NiF_4_-type structured materials with space group I4/mmm^1^. The structure of Sr_2_CoO_4_ consists of corner sharing CoO_6_ octahedra with two-dimension CoO_2_ planes separated by insulating rock-salt layers of SrO. In the past reports, both Sr_2_CoO_4_ single-crystalline thin films and polycrystalline bulks were reported as a metallic ferromagnets with a fairly high Curie temperature (*T*_*C*_) of 255 K^1^,^2^,^3^. The susceptibility data above *T*_*C*_ of Sr_2_CoO_4_ can be well fitted to the Curie-Weiss law χ = C/(T + Θ). The observed value of the effective magnetic moment per Co ion μ_eff_ (μ_B_/Co) is 4.11, which can approximately coincide with that expected for spin only moment of the intermediate-spin (IS) state (t_2g_^4^e_g_^1^, S = 3/2) Co^4+^ (3.87 μ_B_/Co) and be quite different from the values of the low-spin (LS) state (t_2g_^5^e_g_^0^, S = 1/2) Co^4+^ (1.73 μ_B_/Co) and high-spin (HS) state (t_2g_^3^e_g_^2^, S = 5/2) Co^4+^ (5.92 μ_B_/Co)^1^,^4^. Below *T*_*C*_, a cluster-glass state exists in Sr_2_CoO_4_ system^5^. It has been observed that Sr_2_CoO_4_ reveals large magnetic anisotropy where the c-axis is the magnetic easy axis^6^. The coercivity (*H*_*C*_) of Sr_2_CoO_4_ is approximately 2.2~2.5 T at 5 K from polycrystalline sample^1^ and single-crystalline film^2^. It suggests great potential of Sr_2_CoO_4_ for high quality memory applications^7^. The half-metallicity of Sr_2_CoO_4_ has been predicted^6^. Different relationships of the electrical resistivity (*ρ*) versus temperature were observed in polycrystalline Sr_2_CoO_4_ and single-crystalline Sr_2_CoO_4_ film^2^,^4^. The temperature dependence of *ρ* in polycrystalline Sr_2_CoO_4_ exhibits semiconducting characteristics^4^. The *ρ* above *T*_*C*_ for the polycrystalline Sr_2_CoO_4_ can be well fitted by the variable range hopping (VRH) model *ρ* = *ρ*_*0*_exp(*T*_*0*_/*T*)^1/4^. By comparison, in single-crystalline Sr_2_CoO_4_ film, the inter-CoO_2_-plane *ρ* of *c*-axis shows a sharp peak at *T*_*C*_, with a metallic behavior below *T*_*C*_ and a semiconducting behavior above *T*_*C*_^2^. In contrast, the intra-CoO_2_-plane *ρ* of *b*-axis shows metallic characteristics^2^. At low temperature, large negative MR was observed in Sr_2_CoO_4_^1^. The MR reaches a maximum at *H*_*C*_. Therefore, the magnetic and electrical properties of Sr_2_CoO_4_ at low temperature, especially below 5 K, will be rather significant to study. Moreover, certain exceptional and unusual physical phenomenons were observed frequently at low temperature, such as superconductivity, magnetic jump and quantum tunneling^14^,^15^,^16^,^17^,^18^,^19^,^20^.

An interesting phenomenon, a staircaselike behavior which is analogous to resonant quantum tunneling of magnetization, was indeed observed in our Sr_2_CoO_4_ polycrystalline sample below 2.8 K. So far, to our knowledge, this is the first time that the staircaselike behavior was observed in Sr_2_CoO_4_. It may suggest the new application potentials of Sr_2_CoO_4_ in magnetic materials and devices. Thus, systematical experiments are urgently needed to study the exceptional phenomenon of Sr_2_CoO_4_ at low temperature and explore the possibility of the staircaselike behavior for various practical applications. In this work, the magnetic and electrical properties of polycrystalline Sr_2_CoO_4_ were studied below 5 K. The staircaselike behavior was observed in a series of magnetic and electrical curves, such as magnetization versus field (*M-H*) and resistivity versus field (*ρ-H*) curves. Based on the reported researches and explanations on the staircaselike behaviors observed in other materials, the mechanism of the staircaselike behavior in Sr_2_CoO_4_ was discussed in detail.

## Results

**Figure 1:**
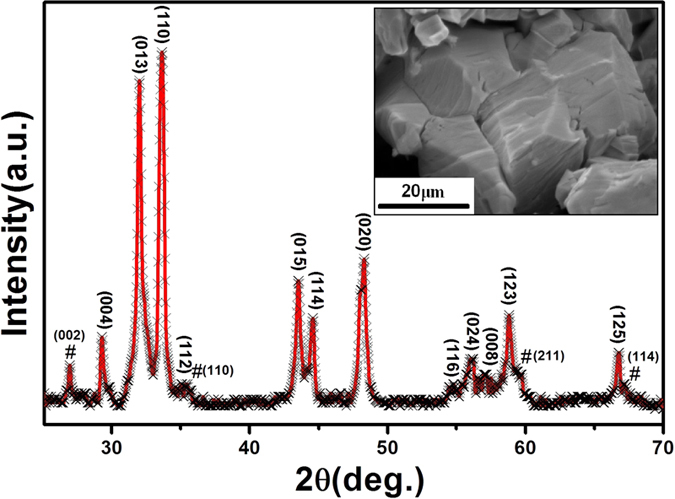
The XRD pattern of polycrystalline Sr_2_CoO_4_ sample. The inset shows the SEM image of polycrystalline Sr_2_CoO_4_ sample.

Figure 1 shows the powder X-ray diffraction (XRD) pattern of polycrystalline Sr_2_CoO_4_ measured at room temperature. The main diffraction peaks of the sample can be fitted well with the XRD profile of Sr_2_CoO_4_ and indexed using the lattice parameters for a tetragonal structure with *a* = 3.8372 Å and *c* = 12.1935 Å. A few additional peaks (marked by #) corresponding to nonmagnetic impurity SrO_2_ can be observed in the pattern. However, this SrO_2_ impurity phase is present in a small amount from the weak intensity of the peaks and has no effect on the magnetic properties of our sample. The inset of Fig. 1 shows the scanning electron microscope (SEM) photograph of Sr_2_CoO_4_. The grains of the sample, with the average size approximately 20 μm, are dense and distribute uniformly.

**Figure 2:**
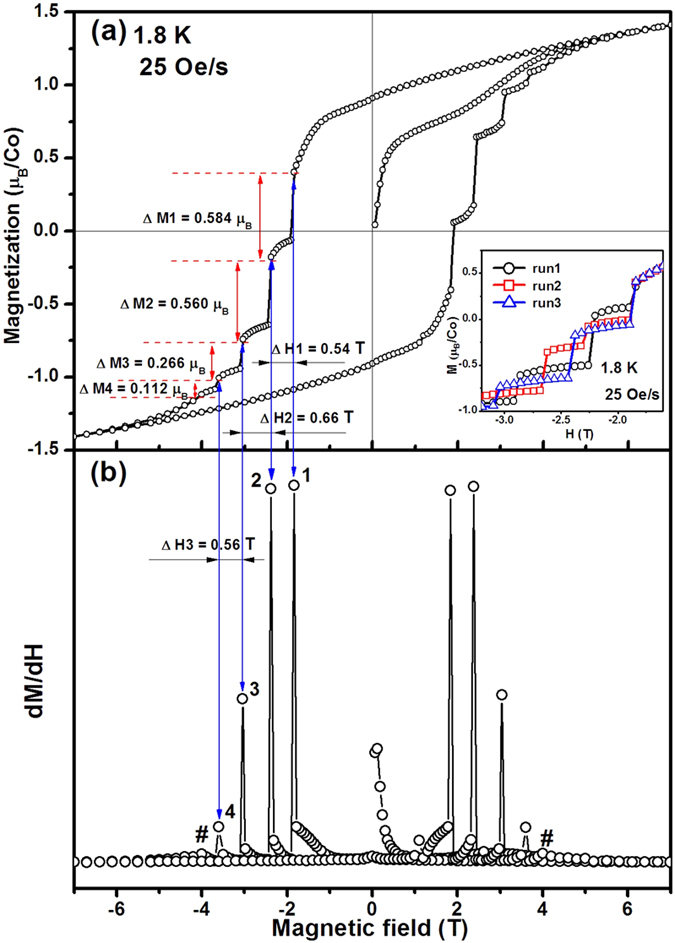
(**a**) Magnetization versus field (*M-H*) curve at 1.8 K with a field sweep rate of 25 Oe/s for the Sr_2_CoO_4_. (**b**) *dM/dH-H* curve at 1.8 K for the Sr_2_CoO_4_. The inset of (**a**) shows the three measurement runs of the same piece of sample obtained under the same measurement condition.

The *M-H* curve of Sr_2_CoO_4_ measured at 1.8 K with a field sweep rate of 25 Oe/s is displayed in Fig. 2(a). The saturation magnetization is 1.02*μ*_*B*_/Co, and the *H*_*C*_ is approximately 1.9 T. The large *H*_*C*_ is caused by high anisotropy in Sr_2_CoO_4_^8^. Most interestingly, unlike the general hysteresis loops, a staircaselike behavior can be observed from the *M-H* loop in Fig. 2(a). The steps on both sides of the hysteresis loop are central symmetry. The span (ΔM) of the four stairs on one side of the loop decreases with the increasing of the applied field (see Fig. 2(a)). The d*M/*d*H* versus field curve (Fig. 2(b)) clearly shows that the four jumps on one side occur at −1.84 T (1), −2.56 T (2), −3.20 T (3), −3.70 T (4), respectively. Moreover, two almost invisible jumps are reflected (see the # in Fig. 2(b)). The inset of Fig. 2(a) shows the three measurement runs from the same piece of sample measured at 1.8 K with a field sweep rate of 25 Oe/s. It can be seen that the steps on these curves of the same piece of sample are obviously misaligned for different measurement runs under the same measurement condition. This result suggests the randomness of the staircaselike behavior in different measurement runs.

**Figure 3:**
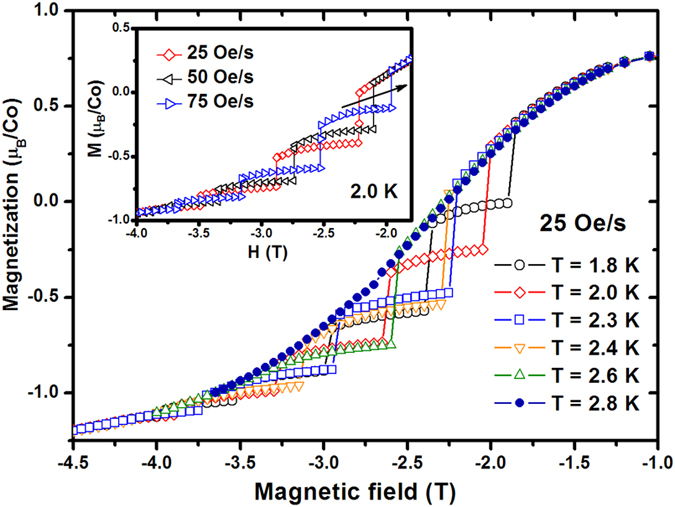
Magnetization versus field (*M-H*) curves for the Sr_2_CoO_4_ sample at different temperatures with the same field sweep rate of 25 Oe/s. The inset shows *M-H* curves for the Sr_2_CoO_4_ at 2 K with different magnetic field sweep rates.

Figure 3 shows the *M-H* curves of Sr_2_CoO_4_ measured at different temperatures. It can be observed that with increasing of the temperature, the quantity of the steps decreases and the positions of the corresponding steps move towards the direction of high field. At 2.8 K, the staircaselike behavior disappears completely. These results suggest that the staircaselike behavior is sensitive excessively to the slight temperature variation. The inset of Fig. 3 shows the *M-H* curves of Sr_2_CoO_4_ measured at 2 K with different magnetic field sweep rates. With the increasing of the sweep rate, the quantity of the steps increases gradually and the positions of the corresponding steps move towards the low field (see the arrow in the inset of Fig. 3). It can be deemed that the staircaselike behavior in Sr_2_CoO_4_ is dependent on the magnetic field sweep rate.

**Figure 4:**
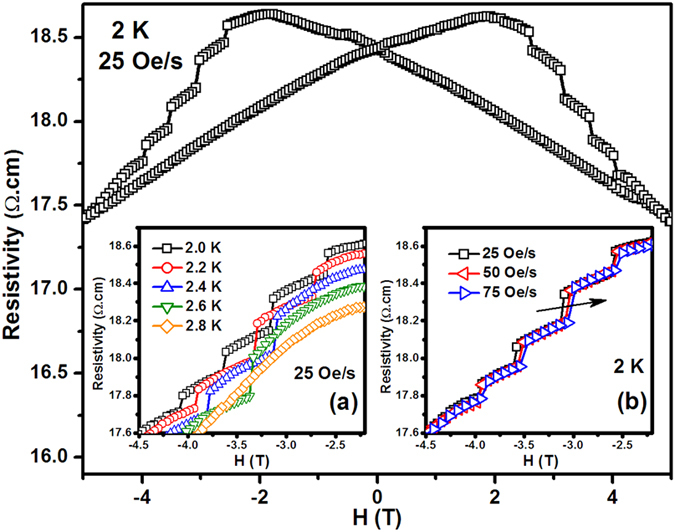
Resistivity versus field (*ρ-H*) curve for the Sr_2_CoO_4_ at 2 K with a field sweep rate of 25 Oe/s. The inset (**a**) shows *ρ-H* curves for the Sr_2_CoO_4_ sample at different temperatures with the same field sweep rate of 25 Oe/s. The inset (**b**) shows *ρ-H* curves for the Sr_2_CoO_4_ sample at 2 K with different magnetic field sweep rates.

Figure 4 shows the *ρ-H* curve of Sr_2_CoO_4_ measured at 2 K. The resistivity reaches a maximum at *H*_*C*_, which is consistent with the previous reports^1^,^2^. This phenomenon can be considered as tunneling MR at domain boundaries. It is attributed to the field suppression of the spin-dependent scattering at domain boundaries^8^. The staircaselike behavior can be also observed from the *ρ-H* curve. The insets (a) and (b) of Fig. 4 show the *ρ-H* curves of Sr_2_CoO_4_ measured at different temperatures and magnetic field sweep rates, respectively. The steps in the inset (a) disappear gradually with the increasing of the temperature. The three *ρ-H* curves in the inset (b) of Fig. 4 are misaligned for different field sweep rates. The positions of the corresponding steps on the *ρ-H* curves also move towards the low field with the increasing of the field sweep rate (see the arrow in the inset (b) of Fig. 4). The steps on the *ρ-H* curves of the same piece of sample are also misaligned for different measurement runs under the same measurement condition (figure not shown). These phenomena are consistent with the above magnetic results of Sr_2_CoO_4_ (see the *M-H* curves of Fig. 3).

## Discussion

Three main characteristics of the staircaselike behavior in Sr_2_CoO_4_ are concluded from the measured results: (i) the positions of the steps are varying in different measurement runs, (ii) the steps only appear at low temperature (T < 2.8 K) and disappear with a slight increase of the temperature, (iii) the steps are dependent on the temperature and field sweep rate. The possible mechanism of the staircaselike behavior will be systematically discussed below.

The similar staircaselike behaviors in hysteresis loops have been also reported in many types of materials, such as Ca_3_Co_2_O_6_^14^,^15^, [Mn_4_]_2_ dimer^16^, Fe_x_Mg_1-x_Cl_2_^21^, PrVO_3_^22^, UGe_2_^23^,^24^, and amorphous Dy-Cu^25^. Simultaneously, different theories have been presented to explain the staircaselike behaviors. The main three theories are resonant quantum tunneling^14^,^15^,^16^,^17^,^18^,^19^,^20^, random field^21^,^22^,^23^,^24^,^25^,^26^,^27^,^28^,^29^,^30^,^31^,^32^,^33^,^34^,^35^, and intrinsic pinning of magnetic domain walls^36^,^37^,^38^.

Resonant quantum tunneling has been applied to systems involving a large number of identical high-spin materials^14^,^15^,^16^,^17^,^18^,^19^,^20^, as in the case of Ca_3_Co_2_O_6_^14^,^15^, and Mn_12_ acetate^20^. Ca_3_Co_2_O_6_ is a type of perovskite material with K_4_CdCl_6_-type structure (an infinite chain-type structure). The analogous steps can be observed from the *M-H* curves of Ca_3_Co_2_O_6_ at low temperature^14^,^15^. The steps are resulted from the transformation and change of the percentage of different magnetism in the materials caused by the applied field at different temperatures^14^. The chain-type structure is the key factor to the staircaselike behavior. The intrachain coupling is ferromagnetic and the interchain coupling is antiferromagnetic. However, Sr_2_CoO_4_ is one type of two-dimensional layer structured compound. Obviously, no chain-type structure exists in Sr_2_CoO_4_. On the other hand, the most important characteristic of the staircaselike behavior in quantum-effect system is that the positions of the steps are temperature-independent below a critical temperature^17^,^18^. The results from the Fig. 3 of Sr_2_CoO_4_ show that the steps in the six *M-H* curves exhibit no similar characteristic of temperature independence. This result indicates that the staircaselike behavior in Sr_2_CoO_4_ is incompatible with resonant quantum tunneling.

The presence of random fields is another explanation that can lead to staircaselike behavior. Under this mechanism, a given domain is flipped by an external field, thus reversing the magnetization of the neighboring domains and finally resulting in an avalanche of flipping domains^21^,^22^,^23^,^24^,^25^,^26^,^27^,^28^,^29^ considering the random field Ising model (RFIM)^31^,^32^,^33^,^34^. Each jump in one curve corresponds to an avalanche process where the spins (of one or more clusters in the polycrystalline Sr_2_CoO_4_) align with the applied magnetic field^26^. The noteworthy characteristic of the steps in this theory is the randomness. The positions of the steps are varying in different measurement runs. Meanwhile, the steps can be only observed at low temperature. The ferromagnetic clusters in Sr_2_CoO_4_ sample play a crucial role for this phenomenon^13^,^26^,^27^,^28^. Below the critical temperature at which the steps are vanished, the ferromagnetic cluster-sizes in the sample increase, and the cluster percolation process yields an increase in the ferromagnetic correlation length with lowering the temperature^26^. The larger cluster-size can result in the bigger avalanche, which gives rise to the distinct jumps. Above the critical temperature, the thermal activation is dominating^26^, and the cluster-size is so small, which can only cause small avalanche. As a consequence, the jumps become sightless, and the hysteresis loop becomes smooth. This type of staircaselike behaviors is dependent on temperature, but independent on field sweep rate. Such an explanation has been proposed in site-diluted metamagnet Fe_x_Mg_1−x_Cl_2_^21^, single crystal antiferromagnet PrVO_3_^22^, single crystalline UGe_2_^23^,^24^, disordered systems such as the amorphous Dy-Cu^25^, polycrystalline CeNi_1−x_Cu_x_^26^,^27^,^28^,^29^, and liquid quenched R_3_Co alloys^30^. All the features of the steps in Sr_2_CoO_4_ are similar to the characteristics of staircaselike behaviors in PrVO_3_^22^, UGe_2_^23^,^24^, and CeNi_1−x_Cu_x_^26^,^27^,^28^,^29^. The other features of the steps, except the dependence of magnetic field sweep rate, can be well explained by the random field theory. The dependence of magnetic field sweep rate may result from the magnetocaloric effect^22^,^35^. The positions of the corresponding steps move to higher field with the decreasing of the sweep rate. It suggests the existence of adiabaticity in Sr_2_CoO_4_. In the adiabatic state, the energy released in the spin reversal process dissipates tardily^35^. With the increasing of the sweep rate, the energy accumulates rapidly and facilitates the reversal of neighboring spins. It results in the sweep rate dependence of the steps. From this point of view, the fundamental reason of the staircaselike behavior in Sr_2_CoO_4_ may be ascribed to an avalanche of flipping domains in terms of the random field theory.

The intrinsic pinning of magnetic domain walls is compatible with the magnetization jumps observed in alloy samples^36^,^37^,^38^. The domain walls motioning inside the ferromagnetic domains depend on the pinning effect introduced by foreign elements and the local crystal fields. The pinning effect can result in the creation of energetic barriers, which influence the magnetization process at low temperature^36^. In the case of EuBaCo_1.92_M_0.08_O_5.5−δ_ (M = Zn, Cu)^37^, Zn^2+^ and Cu^2+^ are the origin of the pinning of the narrow domain walls. When the magnetic field becomes high enough to overcome the pinning effect, the domain walls tend to disappear and the spins of the ferromagnetic domains are all aligned. This type of the staircaselike behaviors strongly depends on the external magnetic field sweep rate. When the magnetic field changes slowly enough, the *M-H* curve becomes normal with no jump^38^. This phenomenon was similar to the result from the inset of Fig. 3 in Sr_2_CoO_4_. In the perfect Sr_2_CoO_4_ crystals, no substituted defect results in the effective pinning. However, here, the saturated moment of the Sr_2_CoO_4_ sample (1.02*μ*_*B*_/Co) is lower than the calculated value (1.97 μ_B_/Co)^2^. Meanwhile, the μ_eff_ of Co ion (4.11 μ_B_/Co) in Sr_2_CoO_4_ is also different from the spin only moments of LS Co^4+^ (1.73 μ_B_/Co), IS Co^4+^ (3.87 μ_B_/Co), and HS Co^4+^ (5.92 μ_B_/Co)^1^,^4^. These results suggest that multiple spin states may exist in our Sr_2_CoO_4_ sample. The interactions between the neighboring IS or HS Co ions (Co(IS or HS)-O-Co(IS or HS)) are antiferromagnetic^39^,^40^, though the ground state of Sr_2_CoO_4_ is ferromagnetic^6^. It means that antiferromagnetism and ferromagnetism are coexistent in Sr_2_CoO_4_, which can lead to multiple magnetic phases. The multiple magnetic phases may result in the intrinsic pinning of magnetic domain walls^36^,^41^,^42^, and further contribute to the magnetization and magneto-transport staircaselike behavior in the Sr_2_CoO_4_.

In summary, layered perovskite compound Sr_2_CoO_4_ polycrystalline sample was synthesized by high temperature and high pressure method. The magnetic and magneto-transport properties of Sr_2_CoO_4_ were studied at low temperature. A staircaselike behavior on *M-H* and *ρ-H* curves was observed in polycrystalline Sr_2_CoO_4_ below 2.8 K. The steps appear with a certain degree of randomness in different measurement runs. The staircaselike behavior is dependent on the temperature and the magnetic field sweep rate. The fundamental reason of the staircaselike behavior can be considered as the presence of random fields, leading to an avalanche of flipping domains. The multiple magnetic phases which can result in the intrinsic pinning of magnetic domain walls, may contribute to the magnetization and magneto-transport staircaselike behavior in the Sr_2_CoO_4_.

## Methods

Polycrystalline sample of composition Sr_2_CoO_4_ was synthesized under high pressure at high temperature. Starting materials of SrO_2_ and Co were well mixed in a molar ratio of SrO_2_ : Co = 2 : 1. The mixture was sealed into a gold capsule. The capsule was first compressed at 6 GPa in a high pressure apparatus (flat-belt-type-high-pressure apparatus, 1500 ton), then heated to 1200 °C for 30 minutes and finally quenched to room temperature followed by releasing of pressure. The crystal structure of the polycrystalline sample was identified by the powder X-ray diffraction (XRD, Rigaku Smartlab3), using Cu-Kα radiation (λ = 1.54184 Å). The morphology of the sample was observed using a scanning electron microscope (SEM). The dc magnetic measurements were investigated using a vibrating sample magnetometer (VSM) integrated in a physical property measurement system (PPMS-9, Quantum Design). The electrical resistivity of the sample was measured with a Quantum Design PPMS-9 system using the standard four-probe ac method.

## Additional Information

**How to cite this article**: Li, Q. *et al*. Magnetization and magneto-transport staircaselike behavior in layered perovskite Sr_2_CoO_4_ at low temperature. *Sci. Rep.* **6**, 27712; doi: 10.1038/srep27712 (2016).
